# New Medical Schools

**Published:** 1848-12

**Authors:** 


*New Medical Schools.*—We learn that a new medical school has lately been organized in Iowa, and, also, another added to the medical school, already existing in Illinois. We have not been favored with circulars from either. A correspondent of the Western Lancet, however, presents the readers of that valuable periodical with several extracts from the circular issued by the latter Institution. That we may do justice to this new enterprise, we transfer to our columns the article referred to, simply remarking that the sentiments contained in the concluding paragraph meet our hearty concurrence:

"On our table lies the Prospectus of this Rock Island Medical School. In this publication the following sentences occur, which sufficiently evince the nature of the enterprise, and the prospects of success which cheer it on.

'That the selection of instructors has been judicious, and that every member of the faculty will sustain himself before the classes *may not be* questioned.' 'They have reasons (not revealed) for believing that a larger number of students *may* assemble at Rock Island than has ever composed *the first class* of any medical school in the Union, and that the resulting graduating class *may be* larger.'

In the prospectus we have the following lines of poetry:

"No pent up Utica contracts our powers,
For the whole boundless UPPER VALLEY's ours."

And again the Prospectus indulges other poetic excursions; though stated in stilted prose.

"No fancy sketch which the imagination can draw can compare with the beauty of the site; and its eligibility, when the Mississippi shall be bridged, can only be rivalled by its beauty. The bridging of the river at this point is so familiarly spoken of by the inhabitants as an improvement and work of art certain to take place before the elapse of many years, that the prediction may not be looked upon as an idle day-dream, that a flourishing Medical School and Hospital *may*, ere long, occupy the very same grounds on which General Taylor was ordered a few years ago to seize and erect a fort in the then wilderness and hostile Indian country. It *may not* come to pass during the presidency of General Taylor, whose policy as far as regards internal improvements, is not very lucidly revealed; neither during the presidency of General Cass, whose letter on river and harbor improvements was certainly laconic; nor yet indeed under the second term of service of the 'northern man with southern principles,' ex-president Van Buren, whose whereabouts on these small questions of river, lake or island improvements is not as well understood of late, as are his views on the great 'Wilmot Proviso question.' But nevertheless at no very remote period, under the administration of some wise president, these things *may* all come to pass on Rock Island, and medicine flourish in this panorama, par excellence, of beauty and promise, where but a few years ago, a lone military post, in the heart of the Indian country, was the solitary herald of civilization."

We have italicised the word *may* in the above and are reminded of the meaning of the word, which is—to be possible, perhaps, by chance, peradventure. There can be no objection to its use in the Prospectus.

After this eulogy of Rock Island the professors come in for a glorification.

The professor of the Theory and Practice has 'views on malarious fevers peculiarly his own, and which differs essentially from the doctrines of the books, and their correctness is proved from the uniform success of his practice, and that of the numerous pupils who have read medicine with him.' Happy pupils! O most excellent master! 'No student ever forgets the doctrines and precepts he receives from this master.' *Quantum sufficit.*

The professor of materia medica has 'a thorough acquaintance with h s subject' and moreover 'his happy arrangement disarms the materia medica of much of the dryness and confusion incident to the old method of teaching still pursued in most of the schools.' *May be* Pereira borrowed this "natural historical arrangement" from Rock Island.

The prospectus promises to receive "obligations" from such students as have no cash.

Such are the aspirations and inspirations—poetic and medical—of the

Rock Island Medical School. Magnificent in conception; exalted in aim; daring and far reaching in destiny!

In all soberness of critical animadversion we lament very much the bad taste and defective judgment, manifested in the prospectus of the Rock Island Medical School. It is a most humiliating spectacle to witness—a professed standard of professional propriety, a fountain head of medical instruction, urging its pretensions, and ushering itself into notice by such self-laudation conveyed in a style of such unseemly grandiloquence." H.

